# Variables Associated with Adherence to Stress Ulcer Prophylaxis in Patients Admitted to the General Hospital Wards: A Prospective Study

**DOI:** 10.15171/apb.2017.009

**Published:** 2017-04-13

**Authors:** Shadi Farsaei, Sajad Ghorbani, Payman Adibi

**Affiliations:** ^1^Department of Clinical Pharmacy and Pharmacy Practice, Isfahan Pharmaceutical Sciences Research Center, Isfahan University of Medical Sciences, Isfahan, Iran.; ^2^Department of Clinical Pharmacy and Pharmacy Practice, Isfahan University of Medical Sciences, Isfahan, Iran.; ^3^Department of Gastroenterology, Integrative Functional Gastroenterology Research Center, Isfahan University of Medical Sciences, Isfahan, Iran.

**Keywords:** Adherence, Academic medical center, Anti-ulcer agents, Clinical practice guideline

## Abstract

***Purpose:*** The dramatic increase in stress ulcer prophylaxis (SUP) prescribing patterns over the past several years has raised concerns regarding to their appropriate utilization. This prospective study attempted to evaluate the trend of adherence to stress ulcer prophylaxis from admission until discharge in non- Intensive care unit (ICU) setting. Additionally, we attempted to find those variables associated with appropriate SUP administration.

***Methods:*** Data collection was performed prospectively to evaluate 195 randomly selected adult patients who received SUP or had indication for that in non-ICU wards of one of the largest referral center in Iran, during 6 months. Adherence was studied according to widely accepted American Society of Health system Pharmacists (ASHP) guideline. Univariate and multivariate logistic regression was also performed to detect associations related to misuse of SUP.

***Results:*** We recognized total inappropriate use of SUP upon admission, during hospital stay and at discharge were somewhat identical at different time points (61%, 80% and 77.4% respectively). On the other hand, since small number of patients experienced SUP underutilization, unfortunately this was not possible to elucidate factors that may have effect on this flawed behavior. However, increasing age was identified to be significant variable in SUP overutilization.

***Conclusion:*** This prospective study highlighted inappropriate overutilization of SUP within non-critically ill patients and found factors which predicted this behavior. Adherence during hospital stay was also calculated for the first time in this study, which was related to SUP adherence upon hospital admission.

## Introduction

Stress ulcer is defined as an acute superficial inflammatory lesions of the gastric mucosa induced by abnormally elevated physiological demand such as sepsis, trauma, burns, and neurologic damage.^1-3^ Although several factors encompass the pathogenesis of stress related mucosal damage (SRMD), but the main reason is ischemia and reperfusion of the vulnerable area.^4^

SRMD predominantly created in the acid-producing area of the stomach (upper body and fundus), and rarely lead to hemodynamically significant gastrointestinal (GI) bleeding in non-critically ill patients.^3^

These mucosal lesions was preliminary observed in central mucosal layer of stomach fundus in 7 critical ill patients after death at 1969.^5^ However, risk of stress ulcer bleeding was high in 1970 (20-30%), but it declined considerably in 1990 (1.5-14%) due to use of prophylaxis medications.^6^ Although, risk of GI bleeding is relatively low in non-critically ill patients (0.41%), but they also could benefit from stress ulcer prophylaxis (SUP) medications.^7^

High rate of SUP prescriptions in intensive care unit (ICU) and general wards necessitated presence of guideline to prevent irrational use of medications.

Therefore, the American Society of Health system Pharmacists (ASHP) was published a guideline for SUP in critically ill patient and suggested two categorical risk factors for SRMD as minor and major risk factors.^8^ This guideline declared a lack of evidence to support the use of SUP in non-critically ill patients with less two minor risk factor for clinically significant bleeding.^9,10^ Although mechanical ventilation, coagulopathy and history of GI bleeding during past year are significant and independent risk factors for stress ulcer in these patients.^1,8-11^

Proton pump inhibitors (PPIs) and histamine 2 receptor antagonists (H2 antagonists) are the most prescribed SUP medications in ICU and general wards of hospital.^3,12^

Previous studies were evaluated the use of SUP in ICU and non-ICU patients because of morbidity and mortality of stress ulcer-related bleeding, the cost of drugs and possible complications associated with SUP administration such as infectious problem and drug-drug interactions related to acid suppression therapy (AST).^13-15^ These studies showed high rate (56-75%) of irrational prescribing of SUP in non-ICU patients.^14^ Although these studies mostly evaluated SUP administration upon admission and at discharge, but data during stay in hospital have been neglected. Therefore, in this study the prevalence of iatrogenic overutilization or underutilization of SUP upon admission, at discharge and during stay in general wards were also evaluated. Additionally, possible factors associated with misuse of AST were reported.

## Materials and Methods

### Methods

It was the cross sectional prospective study performed in the general medical, emergency, and surgical wards at one of the largest teaching hospital in Iran, from September 2014 to March 2015. This mentioned hospital affiliated with residency and fellowship program with around 800 beds that provides medical services to patients from different parts of the country. In this research, the appropriateness of SUP administration was evaluated according to ASHP guideline in adult patients admitted to the medical, surgical and emergency wards. Therefore, patients who received AST for treatment purposes such as GI bleeding, gastroesophageal reflux disease (GERD), peptic ulcer disease (PUD), and dyspepsia did not fulfilled criteria to include in our study. Moreover, patients who transferred from the ICU were excluded.

Patients were randomly selected from different general wards based on the proportion number of admitted patients in each ward. For simple randomization, we assigned a consecutive number to each individual, thereafter, SPSS random number generator was used to produce random numbers.

In addition, an attempt was made to prevent treatment bias induced by physician awareness.

Data collection was performed to gather information regarding demographic characteristics of patients, prescriber service of SUP, admission diagnosis (medical, surgical and trauma), nutritional status, time spent from ward admission to start SUP, related laboratory data, past medical history of GI problem, and duration of admission in hospital wards.

Furthermore, patients were assessed daily for associated risk factors of SRMD, administration of SUP medication (type, dose, route and duration of SUP medications), and GI bleeding during stay in hospital wards.

According to ASHP guideline and recent performed studies, patients who had 1 absolute indications or at least 2 of the relative indications were eligible for SUP administration in non-ICU setting (Table 1).^8,12,16,17^

**Table 1:** Major and minor risk factors for SRMD used in our study according to previous guidelines*

**Major risk factor**
Coagulopathy defined as a platelet count lower than50000 or INR higher than 1.5 or a PTT higher than 2 times the control value
Respiratory failure requiring mechanical ventilation for longer than 48 hours
History of GI ulceration or GI bleeding during past year
**Minor risk factor**
Head trauma with GCS ≤ 10 or spinal cord injury
Burn more than 35 percent BSA
Sepsis
Renal insufficiency
Hepatic failure
Heart failure
Renal or hepatic transplantation
Partial hepatectomy
Use of warfarin
Occult GI bleeding for six or more days
History of use of NSAID more than 3 month
Prolonged NPO status lasting more than 5 days with GI pathology or after major surgery
Glucocorticoid therapy (more than 250 mg hydrocortisone or the equivalent)
Use of heparin with therapeutic dose
Multiple trauma with ISS ≥ 16

* Prophylaxis is recommended in patients with one of the major risk factor or at least two minor risk factors SRMD=Stress related mucosal damage, INR=International normalized ratio, PTT= Partial thromboplastin time, GI=Gastrointestinal, GCS= Glasgow Coma Scale, BSA=Body surface area, NSAID= Nonsteroidal anti-inflammatory drugs, NPO= nil per os (nothing by mouth), ISS=Injury severity score

In our study, SUP administration was assessed upon hospital ward admission, at discharge and during hospital ward stay for both patients who received and did not receive SUP medications.

To evaluate appropriateness of SUP administration during hospital ward stay, we used the below formula to calculate the proportion of days which SUP has or has not been prescribed based on guideline during follow up in our study:

Appropriate percent of SUP prescription = number of days which SUP prescription pattern was in compliance with ASHP guideline / duration of follow up × 100.

According to this formula, appropriateness of SUP administration would be considered if this percent was 80-120%. Meanwhile, percentage more than 120% and less than 80% were defined as overutilization and underutilization, respectively.^18^

Finally, the appropriateness of SUP treatment at hospital discharge was investigated and possible factors related to SUP overutilization were reported. In addition, the cost of non-guideline-based SUP medications’ use was calculated by multiplying the total number of inappropriately utilized medications with the cost of each one.

#### Statistical analysis

Mean and standard error [SE] were used to report quantitative results, and both frequencies and percentages were calculated for qualitative data.

Logistic regression was performed to find the relationship between different variables and SUP overutilization at the beginning and during of hospital stay. Univariate regression analysis was first performed to confirm the importance of these factors and thereafter, multiple logistic regression was conducted to probe the relationship between the previously established risk factors and SUP overutilization. Odds ratio and confidence intervals (95% CI) were considered to report the results of logistic regression model. *P-*value of 0.05 or less was considered significant.

## Results

Of the 204 patients were randomly selected during 6-month period study, 4.4% (9 patients) did not include in our study because they received AST for treatment purposes (7 and 2 patients received AST for dyspepsia and PUD, respectively).

Among 195 patients, 56.4%, 29.8%, and 13.9% were from the medical, surgical and emergency wards respectively, and more than 50% of SUP medications were prescribed by three medical services (internal, surgery and infection) (Table 2).

**Figure 1:**
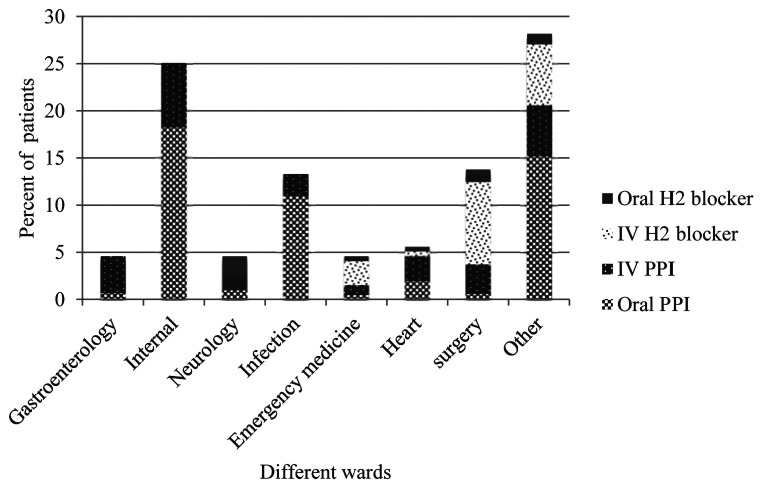
Dosage form of medications administered for stress ulcer prophylaxis based on different wards
IV= Intravenous, PPI= Proton pump inhibitor

The pharmacological agents commonly used upon hospital ward admission and at discharge were PPIs and H2 blockers. Accordingly, PPIs and H2 blockers were prescribed for 117 (75.5%) and 38 (24.5%) of 155 patients upon hospital ward stay, respectively. PPIs were the most frequent medications administered for SUP in patients admitted in gastroenterology, internal, infection and heart wards. Whereas, H2 blockers were the most prescribed AST in neurology, emergency and surgery wards. Moreover, intravenous dosage form of SUP medications were mostly used in gastroenterology, heart, surgery and emergency departments (Figure 1). In addition, PPIs were remained the most prescribed SUP at hospital ward discharge. It should be mentioned that, any combination of PPI and H2 blocker was not administered to the study population.

**Table 2:** Demographic and baseline characteristics of patients completed the study

	**Characteristic**	**Numbers, % or Mean (Std.E)**
**Age**	-	54.2 (1.4)
**Gender**	Male	119 (61)
	Female	76 (39)
**Medical diagnosis**	Medical	137 (70.3)
	Surgical	54 (27.7)
	Trauma	4 (2.1)
**Nutrition**	Oral	163 (83.6)
	NPO	21 (10.8)
	Gavage	7 (3.6)
	TPN	3 (1.5)
	PPN	1 (0.5)
**Prescriber service**	Internal medicine	49 (28)
	Surgery	27 (15.4)
	Infection	26 (14.9)
	Heart	11 (6.3)
	Neurology	9 (5.1)
	Emergency medicine	9 (5.1)
	Gastroenterology	9 (5.1)
	Orthopedist	6 (3.6)
	Neurologist	6 (3.6)
	Oncology	5 (2.9)
	Others	18 (10.3)

Std.E=standard error, NPO=nil per os (nothing by mouth), TPN=total parenteral nutrition, PPN=partial parenteral nutrition

The time lasted from hospital ward stay to begin SUP administration was 0.3 ± 0.1 day.

Moreover, duration of patient follow up and taking SUP in the hospital ward were 8.0 ± 0.5 and 7.4 ± 0.5 days, respectively.

Our results revealed inappropriate use of SUP upon admission, during hospital stay and at discharge were 61%, 80% and 77.4%, respectively.

Among included patients, 155 received SUP upon hospital ward admission, 109 (70.33%) patients did not fulfill the criteria to receive SUP. In addition, 10 out of 40 (25%) patients did not receive SUP properly upon admission while they had clear or valid indication.

We also assessed appropriate percent of SUP prescription during hospital ward stay (according to declared formula in method). Therefore, SUP prescriptions were considered over-utilized in 137 of 195 (70.3%) patients in the average of 6.6 ± 0.5 days. However, only in 19 of 195 (9.8%) patients under-utilization were occurred for 3.5 ± 1.3 days. On the other word, SUP was prescribed properly for 28 of 175 (16.0%) patients who received SUP, and 9 of 20 (45.0%) patients who did not receive SUP during hospital ward stay. In general, results revealed SUP was prescribed appropriately for 37 of 195 (19.0%) patients during hospital stay.

At the end, SUP was continued appropriately in 10 of 22 (45.5%) patients at hospital discharge. Moreover, 139 of 173 (80.3%) patients did not receive SUP properly according to guideline (Table 3).

**Table 3:** Frequency of eligible patients for stress ulcer prophylaxis

**Risk factor**	**Number (% from 195 included patients )**	
**Major ASHP criteria**	**Upon ward admission**	**At hospital discharge**
Coagulopathy	29 (16.6)	16 (9.2)
Mechanical ventilation for longer than 48 hours	-	-
History of GI ulceration or GI bleeding during past year	11 (6.3)	11 (6.3)
Patients with at least 1 of the major ASHP criteria ^*^	38 (19.5)	28 (14.3)
**Minor ASHP criteria**		
Head trauma or spinal cord injury	5 (2.5)	4 (2.1)
Burn more than 35 percent BSA	-	-
Sepsis	3 (1.5)	3 (1.5)
Renal insufficiency	23 (11.8)	18 (9.2)
Hepatic failure	3 (1.5)	3 (1.5)
Heart failure	9 (4.6)	9 (4.6)
Use of warfarin	10 (5.1)	3 (1.5)
History of use of NSAIDs more than 3 month	3 (1.5)	3 (1.5)
Prolonged NPO status lasting more than 5 days with GI pathology or after major surgery	1 (0.5)	5 (2.5)
Glucocorticoid therapy ^†^	16 (8.2)	2 (1.03)
Use of heparin with therapeutic dose	18 (9.8)	7 (3.6)
Patients meeting at least 2 of the minor ASHP criteria *	70 (35.9)	49 (25.1)
**Number (%)**		
Patients received stress ulcer prophylaxis when it was not indicated	109 (70.3) ^‡^	12 (54.5) ^††^
Patients did not receive stress ulcer prophylaxis when it was indicated	10 (25) ^§^	34 (19.6) ^‡‡^

*This number is less than the sum of total patients meeting ASHP major criteria because some patients had more than 1 criterion † More than 250 mg hydrocortisone or the equivalent ‡Among 155 patients who received SUP §Among 40 patients who not received SUP †† Among 22 patients who received SUP ‡‡ Among 173 patients who not received SUP ASHP= American society of health-system pharmacists, BSA=Body surface area, NSAID= Nonsteroidal anti-inflammatory drugs, NPO= nil per os (nothing by mouth), GI=Gastrointestinal

**Figure 2:**
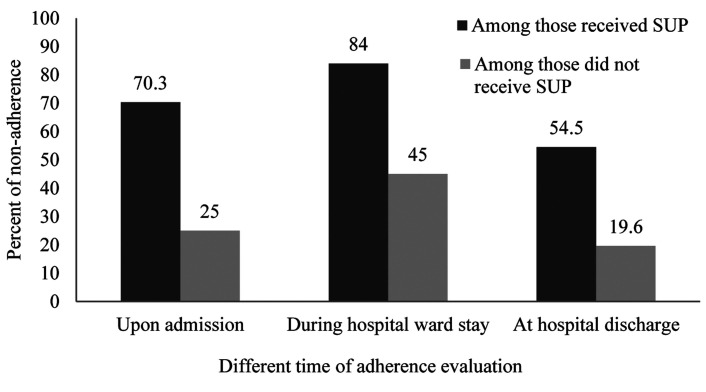
Overview of non-adherence to stress ulcer prophylaxis in general wards SUP= Stress ulcer prophylaxis

Consequently, inappropriate administration of SUP upon hospital ward admission, at discharge and during hospital stay were summarized in Figure 2. Non-adherence was higher among those received SUP than who did not receive it in different time of evaluation. However, inappropriate use of SUP increased during hospital stay than upon admission, but it reached the lowest percent at hospital discharge.

The total cost of non-guideline-based SUP medications’ use was $3,500 for 204 patients evaluated in our study. It should be mentioned that intravenous pantoprazole and ranitidine were considered for 70% and 14% of this cost, respectively.

Among 195 patients, only 1 (0.51%) patient experienced GI bleeding during follow up. He admitted to hospital with diagnosis of myocardial infarction and past medical history of GI bleeding. In addition, mortality rate in our study was 11 of 195 (5.64%) patients.

### Factors associated with inappropriate SUP administration

All variables were analyzed to evaluate factors affecting adherence to SUP practice. Since small number of patients experienced SUP underutilization during hospital ward stay, unfortunately this was not possible to elucidate factors that may have an effect on this behavior. Therefore, variables that may have dictated overutilization were summarized in Table 4.

According to univariate analysis, a significant increase in the risk of wrong decision to start SUP was observed in female patients (*p* = 0.002). Although it must be noted that the number of minor and major risk factors were not significantly different when variable of gender was studied (*p* = 0.81 and *p* = 1.00 respectively).

**Table 4:** Variables affecting SUP overuse at admission and during hospital stay

**-**	**Upon hospital admission** ^µ^					**During hospital stay** ^Ω^				
	**Univariate regression**		**Multivariate regression**			**Univariate regression**		**Multivariate regression**		
	*p*-value	OR	*p*-value	OR	95% CI	*p*-value	OR	*p*-value	OR	95% CI
**Age**	<0.001	1.013	0.024	1.011	1.001-1.020	< 0.001	1.024	0.003	1.018	1.006-1.029
**Female gender**	0.002	2.471	0.372	1.369	0.687-2.727	< 0.001	3.643	0.911	1.044	0.490-2.223
**Duration of hospital stay**	0.002	1.053	0.944	1.002	0.959-1.046	< 0.001	1.137	0.170	1.041	0.983-1.103

All p-values calculated by binary logistic regression test µ: Among patients who received SUP, 46 patients were SUP candidate and in 109 SUP were over-utilized Ω: Among patients who received SUP, 28 had appropriate adherence and in 137 SUP were over-utilized OR=odds ratio, CI=confidence interval.

Moreover, older age and longer duration of hospital stay were shown to be a major predictor of SUP overuse (*p* = < 0.001 and *p* = 0.002, respectively). However, only increased age remained statistically significant when multivariate model was developed at hospital admission (*p* = 0.024, OR = 1.011, 95% CI = 1.006-1.029).

It should be mentioned that factors affecting SUP overuse were similar upon hospital admission and during hospital stay in both univariate and multivariate analyses.

## Discussion

Although current medical care could decrease the prevalence of stress-related GI bleeding by 17% in recent years, but routine administration of SUP over the years in most non-ICU hospitalized patients has emerged an important challenge in health system.^3,6^ This may be related to less defined risk factors which could identify high risk patients who would benefit from SUP use in non-ICU hospitalized patients.^6,19^ However, it should be mentioned that these risk factors were well studied and determined in ICU patients.^8,20^ Despite this limitation, similar to previous conducted studies, we used the modified version of ASHP risk factors as the guidance to evaluate appropriateness of SUP administration in non-ICU patients.^3^

Our results revealed SUP was prescribed in the majority proportion of included patients upon hospital admission; despite, most of them were considered inappropriate according to guideline. This unsuitable pattern of SUP prescription also remained at discharge. Unfortunately, this dramatic finding is comparable with recent literature review which indicates high percent of patients received SUP improperly upon hospital admission and at discharge (22-93% and 44-88%, respectively).^3,10,12,16,17,21^ High prevalence of irrational prescribing in some institutes necessitated clinical pharmacist intervention, which could improve the prescription pattern of SUP administration in certain hospital wards.^22,23^

Different study design (retrospective, cross-sectional and prospective) and institutes where studies were conducted justified this wide range of non-adherence to SUP guideline in previous studies. However, this rate of adherence is relatively higher in ICU patients who have more definitive risk factors to initiate SUP medications.^18^ In addition, teaching or nonteaching setting of study could have an effect on inappropriate SUP prescription. Recent studies revealed SUP usage in academic centers were more compliant with the guidelines versus nonacademic hospitals.^17,24,25^

It seems that evaluation of adherence upon admission and at discharge are inadequate to give a complete view of rationale prescribing of SUP medications. Accordingly, we also calculated adherence during hospital stay and unfortunately we found small number of physicians adhered to the SUP guideline during hospitalization which is similar to data related to hospital admission. On the other word, inappropriate beginning of SUP was continued during hospital stay in the same manner. These findings showed first day adherence could be a good predictor of adherence during hospital stay.

We should mention that since we did not find any previous report to calculate this adherence, we theorized that patients who received 80-120% of SUP medications appropriately were adhered to the guideline. Moreover, we did not find the appropriate time needed to pass after risk factors were resolved to stop SUP medications. Therefore, we considered patients ineligible to continue SUP when the appropriate indications were disappeared. However this decision is somewhat more difficult in ICU setting where patients are at increased risk of stress induced ulcers and consequent bleeding.^26^

In addition, we attempted to elucidate some factors that may be associated with adherence to SUP prescriptions. Nonetheless, because of low percentage of underutilized patients, we only could find factors that predicted overutilization.

Although previous conducted studies identified some predictive factors for AST overuse, but data is not enough especially in developing countries and there is controversy among published results.^3,10,21,27-30^

We identified similar risk factors of AST overuse both upon hospital admission and during hospital stay. Increased age, duration of hospital stay, and female gender were suggested to be significant factors that could affect on AST overuse in our results. Nevertheless, only increased age remained significant variable in multivariate analysis among the mentioned factors, while both duration of hospital stay and gender became a trend.

The rational of more AST overuse during longer hospital stay is understandable. Since the sicker patients required longer hospital stay and more medical care, so unconscious physicians may be encouraged to begin SUP to prevent more complication of GI bleeding. It is surprising that, Issa et al^3^ also has identified similar factors contributing in SUP overuse. They revealed hospital stay as the only significant factors affecting SUP overuse in multivariate analysis whereas, age and male gender became a trend. However, not only other observational studies did not identify significantly shorter duration of hospital stay in patients who received SUP appropriately, but one of this studies suggested the negative trend between hospital stay and AST overuse.^21,30^

Increased age was another significant factor impacting AST overuse in our study. It is reasonable prediction that unconsciousness physicians considered SUP for advanced age patients predisposing to more medical problems. Other performed studies also confirmed our results.^3,27^ A retrospective medical chart review suggested increasing patient age as a clear variable in AST overuse.^31^ The results of another retrospective review noted intravenous PPI was overused in inpatients with age ≥ 65 years old.^10^

The reason that female gender was considered as predictive factor for AST overuse in our study is unclear. Furthermore, some previous studies released data showing otherwise, they suggested male gender as associated variable that could significantly predict AST overuse.^21,30^ Although other studies found that female gender was independently associated with inappropriate AST prescribing in both primary care and hospitalized patients.^28,29^ Review of published studies revealed conflicting results regarding the effect of gender on SUP adherence, but none of them could explain the logic of these inconsistence.

Not surprisingly, overuse of AST is not devoid of side effects. In fact, an increased risk of developing clostridium difficile-associated diarrhea and possibly nosocomial pneumonia may be associated with AST use, which is greater with PPIs.^32,33^ However, more evaluation is required to determine this causality.^33^ Furthermore, numerous potential interactions such as drug-drug, drug-nutrient and drug-test interactions have been described with variety of mechanism.^34,35^

Therefore, this inappropriate practice of SUP prescribing has substantial burden for both patients and health care providers.

Some limitations of the present prospective study need to be noticed. A relatively small number of studied patients might not be an appropriate sample to completely represent the population of admitted patients in non-ICU wards of this large hospital. Moreover, since mostly residents rather than attending physicians decide to start and continue SUP in academic center, it is difficult to extrapolate the result of one academic center to other hospitals. Not determining the possible complications of SUP administration (e.g., pneumonia or CAD) in the study population can be considered as the other limitation of this study.

However, the prospective design of our study is the strength point of that, because missing data is highly unlikely. Moreover, our results showed variables associated to SUP misuse in one of the largest hospital in a developing country, which give a chance to compare results with other conducted studies. Our results also give wide viewing about SUP adherence upon admission, during hospital stay and upon discharge and could use as a guide for appropriate future practice of physicians. Possible interventions such as application of internal guideline, preparing order template which contains indication of SUP administration, stop orders, automatic switch order from PPI to H2 blocker, education of residents and nursing staff and implementation of clinical pharmacists' activities can be helpful to decrease unnecessary AST use.

## Conclusion

We found high inappropriate SUP utilization according to ASHP guideline upon admission, during hospital stay and at discharge (61%, 80% and 77.4%, respectively). This prospective study highlighted unsuitable overutilization of SUP within non-critically ill patients and found factors which predicted this behavior such as older age and longer duration of hospital stay. Adherence during hospital stay was also calculated for the first time in this study and we found it related to SUP adherence upon hospital admission. It is clear that AST overuse may cause significant adverse effects and imposes unnecessary financial burden on both patients and hospital. Therefore, controlled policies require to manage inappropriate SUP administration. Further studies may also need to evaluate variables associated with SUP overuse in the outpatient setting.

## Acknowledgments

We would like to thank staff members of Alzahra Hospital for their cooperation (general support). It also should be declared that this study was financially supported by Isfahan University of Medical Sciences.

## Ethical Issues

The study protocol was approved by the ethical committee of Isfahan University of Medical Sciences.

## Conflict of Interest

The authors have no conflicts of interest to declare.
